# Intermittent High-Grade Atrioventricular Block as a Presenting Sign of Left Ventricular Noncompaction

**DOI:** 10.1016/j.jaccas.2026.107647

**Published:** 2026-03-25

**Authors:** Mashkurul Haque, Aubriannah Larson, Ola Abdelkarim, Vikram Sharma

**Affiliations:** a Department of Internal Medicine, University of Iowa Health Care, Iowa City, Iowa, USA; b Division of Cardiovascular Medicine, Department of Internal Medicine, University of Iowa Health Care, Iowa City, Iowa, USA

**Keywords:** cardiac magnetic resonance, cardiomyopathy, imaging, left ventricle

## Abstract

**Background:**

Left ventricular noncompaction is characterized by excessive trabeculation and is variably associated with heart failure, arrhythmias, thromboembolism, and conduction disease.

**Case Summary:**

A 37-year-old man with palpitations and chest discomfort had intermittent high-grade (Mobitz II) atrioventricular block on event monitoring (3.7-second pause). Cardiac magnetic resonance revealed apicolateral hypertrabeculation with a diastolic noncompacted:compacted ratio of 3.5 without late gadolinium enhancement; transthoracic echocardiography showed preserved ejection fraction (60%). Electrophysiology recommended longitudinal rhythm surveillance with an implantable loop recorder; pacemaker was deferred. Genetic testing was advised but not authorized by insurance.

**Discussion:**

Intermittent high-grade atrioventricular block can be an initial manifestation of left ventricular noncompaction with preserved systolic function. Recognition of this association supports advanced imaging in unexplained conduction disease in young adults and favors loop-recorder–guided surveillance with multidisciplinary follow-up, including genetics.

**Take-Home Messages:**

Unexplained high-grade atrioventricular block in a young adult should prompt advanced structural evaluation—including cardiac magnetic resonance—to exclude left ventricular noncompaction and other cardiomyopathies. Preserved ejection fraction and absent late gadolinium enhancement can coexist with clinically relevant conduction disease in left ventricular noncompaction. Implantable loop recorder–guided surveillance enables individualized timing of device therapy and complements genetic evaluation and cascade family screening.

## Graphical Abstract

**Figure:**
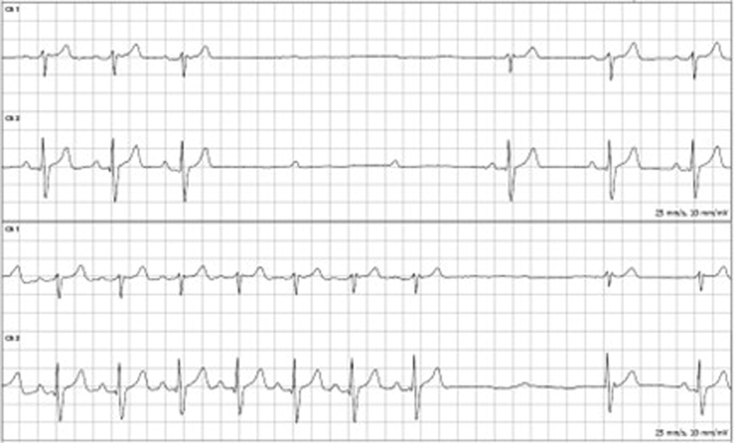

## Case Presentation

**Figure 1:**
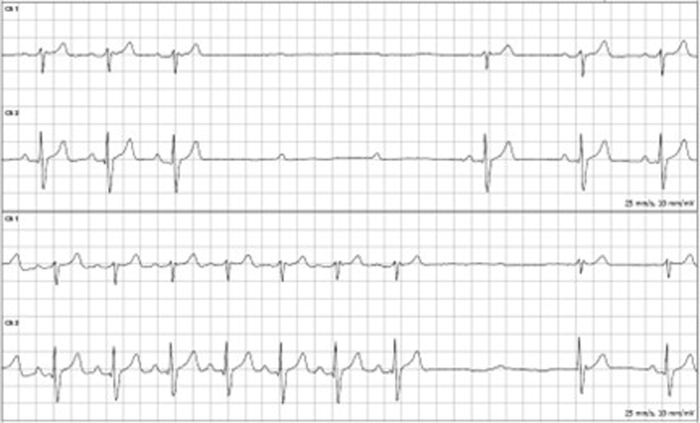
Event Monitor Tracing Demonstrating High-Degree Atrioventricular Block With Atrioventricular Dissociation P waves continue at a regular interval without corresponding QRS complexes, indicating nonconducted atrial activity. Ventricular activity resumes without restoration of atrioventricular synchrony.

A 37-year-old man with attention-deficit/hyperactivity disorder and depression presented for evaluation after a 30-day ambulatory event monitor captured intermittent high-grade (Mobitz II) atrioventricular (AV) block with the longest pause of 3.7 seconds while awake (Figure 1). He initially reported intermittent palpitations and stress-related chest discomfort without syncope. On the day of the recorded event (June 1, 2025, ∼09:54 am), he was performing routine activities and subsequently presented to the emergency department for further evaluation.

**Take-Home Message**

• Unexplained high-grade atrioventricular block in a young adult should prompt advanced structural evaluation—including cardiac magnetic resonance—to exclude left ventricular noncompaction and other cardiomyopathies.
• Preserved ejection fraction and absent late gadolinium enhancement can coexist with clinically relevant conduction disease in left ventricular noncompaction.
• Implantable loop recorder–guided surveillance enables individualized timing of device therapy and complements genetic evaluation and cascade family screening.

Baseline 12-lead electrocardiography demonstrated sinus rhythm with right bundle branch block and left anterior fascicular block. Transthoracic echocardiography earlier in the year showed normal chamber sizes and preserved left ventricular ejection fraction (LVEF) (60%) without regional wall motion abnormalities.

In a young adult presenting with new-onset high-grade AV block, the differential diagnosis included both intrinsic conduction system disease and structural or infiltrative cardiomyopathies. Reversible causes such as electrolyte derangements, thyroid dysfunction, medications, Lyme carditis, and myocarditis were considered but were not supported by history, laboratory testing, or inflammatory markers. The presence of baseline right bundle branch block with left anterior fascicular block raised concern for His-Purkinje system involvement, including idiopathic progressive conduction disease or infiltrative conditions such as sarcoidosis or amyloidosis; however, the absence of systemic features and preserved ventricular size and function made these less likely. Ischemic conduction disease was considered improbable given the patient's age, risk profile, and lack of ischemic symptoms. The possibility of a genetic or inherited cardiomyopathy was also considered, prompting further evaluation with cardiac magnetic resonance (CMR) to assess for noncompaction, fibrosis, or other structural substrates underlying the conduction disturbance.

**Figure 2:**
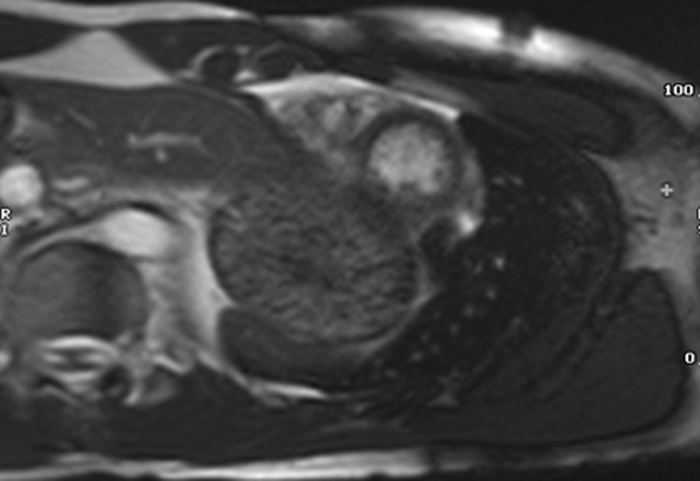
Short-Axis End-Diastolic Cardiac Magnetic Resonance Image Demonstrating Apical and Lateral Hypertrabeculations The noncompacted:compacted myocardial ratio exceeds 3.5 in the apicolateral segment, consistent with left ventricular noncompaction diagnostic criteria.

**Figure 3:**
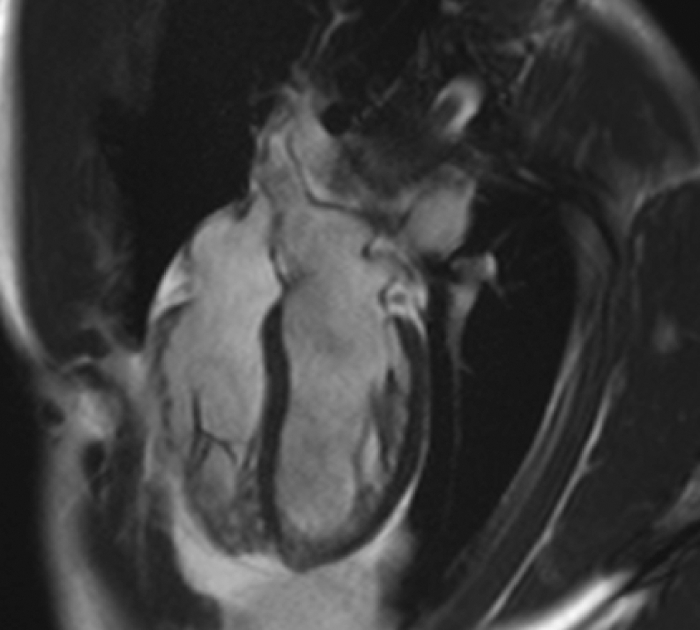
4-Chamber Cine Still From Cardiac Cardiac Magnetic Resonance Illustrating Deep Intertrabecular Recesses in the Apex and Lateral Wall of the Left Ventricle, Typical of Noncompaction Morphology

CMR performed during the index evaluation showed prominent trabeculations in the apical and lateral left ventricular segments (Figures 2 and 3). The diastolic noncompacted:compacted (NC/C) ratio measured 3.5 in the apicolateral wall (meeting established CMR diagnostic criteria) with a LVEF 56% and no late gadolinium enhancement (LGE).

Multidisciplinary assessment by cardiomyopathy and electrophysiology services concluded that, given preserved systolic function, absence of syncope, and the intermittent nature of the conduction abnormality, permanent pacing could be deferred in favor of longitudinal rhythm surveillance with an implantable loop recorder (Boston Scientific LUX-Dx II+). He was counseled on genetic testing via a comprehensive cardiomyopathy/arrhythmia panel with cascade screening if a pathogenic or likely pathogenic variant was identified; authorization was denied by insurance. He later relocated to Georgia and continued follow-up at a tertiary center.

## Discussion

Left ventricular noncompaction (LVNC) is characterized by a bilayered myocardium with deep intertrabecular recesses and disproportionately prominent trabeculae. On CMR, a diastolic NC/C ratio >2.3 is commonly used to differentiate pathologic LVNC from physiological trabeculation^1^; echocardiographic frameworks (eg, Jenni/Oechslin) emphasize a thin compacted epicardial layer with a thicker noncompacted endocardial layer and typical segmental distribution.^2^ Our patient's apicolateral NC/C of 3.5 and preserved LVEF (56%) meet CMR criteria and align with the expected distribution.

Not all excessive trabeculation implies disease. Contemporary expert guidance underscores that, in adults, trabeculation extent alone has not shown independent prognostic impact once ventricular function and underlying myocardial disease are accounted for; imaging should be interpreted in clinical context.^3^^,^^4^ In this case, the absence of LGE argues against overt scar; however, the presentation with intermittent high-grade AV block points to clinically meaningful conduction involvement requiring surveillance.

Conduction disease and arrhythmias are well recognized in LVNC. Reviews and series document associations with bundle-branch block, high-grade AV block, supraventricular tachyarrhythmias, and monomorphic ventricular tachycardia, which carry implications for sudden death risk and device therapy in selected phenotypes.^5^^,^^6^ The electrophysiology-guided decision to defer pacemaker implantation in favor of implantable loop recorder surveillance is consistent with phenotype-directed care in an asymptomatic patient without sustained bradycardia or syncope.

Genetically, LVNC is heterogeneous. A ClinGen-style synthesis identified 189 reported genes, of which only a minority meet definitive or moderate evidence thresholds; most signal converges on sarcomeric (eg, *MYH7*, *TTN*), cytoskeletal (eg, *LMNA*), and channel/conduction genes (eg, *SCN5A*, *HCN4*).^7^ Of note, *HCN4* variants can present with sinus bradycardia plus LVNC, supporting a mechanistic link between conduction dysfunction and trabecular phenotype.^8^ Cancer-predisposition genes such as *CHEK2* and *NF1* are not established causes of LVNC; when present in relatives, they typically warrant separate oncogenetic counseling rather than substitution for a comprehensive cardiomyopathy/arrhythmia panel. Accordingly, a broad panel with cascade screening of first-degree relatives is appropriate when a pathogenic/likely pathogenic variant is identified.^4^^,^^7^

Adult LVNC outcomes are heterogeneous and appear largely modulated by LVEF, ventricular arrhythmias, and fibrosis. A 2024 adult systematic review and meta-analysis reported higher detection by CMR than echocardiography and identified reduced LVEF and ventricular tachycardia as key adverse prognostic markers.^9^ Although LGE is the most validated tissue marker of adverse events across cardiomyopathies, its absence does not obviate rhythm-related risk; ongoing implantable loop recorder surveillance in this conduction-predominant presentation is therefore prudent. (Robust multicenter dilated cardiomyopathy data illustrate the prognostic signal of LGE for death/transplant/left ventricular assist device and arrhythmic events,^10^ and support using LGE contextually when interpreting LVNC CMR.)

This case underscores 3 points. First, unexplained high-grade AV block in a young adult should prompt structural evaluation for cardiomyopathy—including LVNC—with CMR. Second, preserved ejection fraction and absent LGE can coexist with clinically relevant conduction disease. Third, implantable loop recorder–guided surveillance enables individualized timing of device therapy and complements genetic evaluation and family screening.

## Funding Support and Author Disclosures

The authors have reported that they have no relationships relevant to the contents of this paper to disclose.
